# Supplementary material for: Unrecognized and Unreported Concussions Among Community Rugby Players
Source: Sports (Basel). 2025 Aug 20;13(8):278. doi: 10.3390/sports13080278 (PMC12390137; doi:10.3390/sports13080278)
Supplement: Supplementary file 1 [file sports-13-00278-s001.zip › sports-3774712-supplementary.pdf]

Supplemental Material

Supplemental Methods: Gilbert Injury History Questionnaire – Questions Used for Diagnosed Concussin, Concussion Nondisclosure, and Unrecognized Concussion

4. Have you ever suffered a concussion? YES NO
   a. If yes, how many? ____________________
   b. If yes, approximately when were they? (Month and year to the best of your memory)
   ____

5. Did you ever suffer a concussion and not tell anyone? YES NO
   a. If yes, why? (check all that apply)
   ____ 1. Did not think it was serious
   ____ 2. Did not know it was a concussion
   ____ 3. Did not want to be pulled out of the game/practice
   ____ 4. Did not want to be pulled from future games/practice
   ____ 5. Did not want to let your teammates down
   ____ 6. Would have if it was a less important game/practice
   ____ 7. Other:____________________

20. Have you ever been hit so hard you lost your memory while playing sports? YES NO
   a. If yes, how many times? __________ How many were diagnosed as a concussion? _________
   b. If yes, did you tell your coach, athletic trainer, or parent? Which one(s)?

12. Have you ever been "knocked silly/seen stars" (confused/disoriented) while playing sports? YES NO
   a. If yes, how many times? _______ How many were diagnosed as a concussion? _________
   b. If yes, did you tell your coach, athletic trainer, or parent? Which one(s)?

Supplemental Table S1. Demographics by Concussion History

	**Total**	**No History of Concussion**	**History of Concussion**	**p-value**	**Effect Size**
	N=1,037	N=347	N=690		
**Age, years**	31.6 (11.3)	30.0 (10.9)	32.5 (11.4)	<0.001	0.225
**Sex**				0.52	0.042
Male	612 (59.0%)	200 (57.6%)	412 (59.7%)		
Female	425 (41.0%)	147 (42.4%)	278 (40.3%)		
**Position**				0.49	0.045
Forward	631 (60.8%)	206 (59.4%)	425 (61.6%)		
Back	406 (39.2%)	141 (40.6%)	265 (38.4%)		
**Years Rugby Played**	10.1 (8.1)	8.6 (7.0)	10.9 (8.5)	<0.001	0.290
**Number of Diagnosed Concussion**	3.0 (2.6)	N/A	3.0 (2.6)	N/A	
**Intentionally Unreported Concussion**				<0.001	0.999
No	701 (67.6%)	325 (93.7%)	376 (54.5%)		
Yes	336 (32.4%)	22 (6.3%)	314 (45.5%)		
**Potentially Unrecognized Concussion**				<0.001	0.655
No	599 (57.8%)	270 (77.8%)	329 (47.7%)		
Yes	438 (42.2%)	77 (22.2%)	361 (52.3%)		

Note: Cohen's d interpreted as small (0.2), moderate (0.5), or large (0.8).

Supplemental Table S2. Demographics by Unreported Concussion

	Total	No Unreported Concussion	History of Unreported Concussion	p-value	Effect Size
	N=1,037	N=701	N=336		
**Age, years**	31.6 (11.3)	31.0 (11.0)	32.9 (11.7)	0.009	0.171
**Sex**				0.24	0.077
Male	612 (59.0%)	405 (57.8%)	207 (61.6%)		
Female	425 (41.0%)	296 (42.2%)	129 (38.4%)		
**Position**				0.54	0.041
Forward	631 (60.8%)	422 (60.2%)	209 (62.2%)		
Back	406 (39.2%)	279 (39.8%)	127 (37.8%)		
**Years Rugby Played**	10.1 (8.1)	9.5 (7.7)	11.4 (8.6)	<0.001	0.226
**Diagnosed Concussion**				<0.001	0.759
No	347 (33.5%)	325 (46.4%)	22 (6.5%)		
Yes	690 (66.5%)	376 (53.6%)	314 (93.5%)		
**Number of Diagnosed Concussion**	2.0 (2.6)	1.1 (1.5)	3.8 (3.3)	<0.001	1.067
**Potentially Unrecognized Concussion**				<0.001	0.711
No	599 (57.8%)	481 (68.6%)	118 (35.1%)		
Yes	438 (42.2%)	220 (31.4%)	218 (64.9%)		

Note: Cohen’s d interpreted as small (0.2), moderate (0.5), or large (0.8).
